# Implementation science: why should we care?

**DOI:** 10.5195/jmla.2024.1919

**Published:** 2024-07-01

**Authors:** Frances Chu

**Affiliations:** 1 frances.chu@providence.org Medical Librarian, Providence Swedish, 747 Broadway, Seattle, WA

**Keywords:** Implementation Science

## Abstract

There is a 17-year gap between the publication of research which proves an intervention is efficacious and effective and the implementation of that same intervention into practice [1]. In behavioral health, only 14% of successful interventions are integrated into actual practice [2]. As such, Implementation Science is envisioned to address the research to practice gap. This research methodology becomes important as it looks to investigate how to get interventions to become embedded in practice and de-implement unproven or disproven interventions that may be harmful and/or ineffective for patients.

The aim of this commentary is to raise awareness of health sciences librarians/information specialists about this research arena and encourage health sciences librarians to envision how they could be involved in implementation science projects and teams or even use implementation science in their practice.

## WHAT IS IMPLEMENTATION SCIENCE?

Implementation science was described in 2006 by Eccles and Mittman [3] and has been defined as “the scientific study of methods to promote the systematic uptake of research findings and other evidence-based practices into routine practice, and hence, to improve the quality and effectiveness of health services or care” [3]. Other synonyms and similar terms are dissemination and implementation, implementation research, knowledge transfer, knowledge translation, knowledge integration, research utilization, improvement science, etc. [4]. The goal of implementation science focuses on the processes to introduce and embed solutions to problems into a health system or community [5]. Peters [5] stated, “the intent is to understand what, why, and how interventions work in “real world” and to test approaches to improve them”. In other words, this research area is focused on strategies and tactics to enhance adoption, implementation, scaling up, and sustainability of evidence-based interventions which could be programs, practices, principles, procedures, products, pills and/or policies that will change health behaviors, health outcomes or health environments [6].

## WHAT IS THE DIFFERENCE BETWEEN IMPLEMENTATION SCIENCE RESEARCH AND EFFECTIVENESS/EFFICACY RESEARCH?

Implementation science must be distinguished from effectiveness/efficacy research. Effectiveness/efficacy research typically has the goal of investigating interventions for specific health problems. Efficacy studies of interventions answer the question of whether an intervention could work under strict, rigorous conditions while effectiveness (also known as pragmatic) studies of interventions seek to investigate whether the intervention will work in real-world settings. The outcomes of efficacy and effectiveness studies focus on patient outcomes who are typically the targets of the intervention. Implementation studies are focused on how to make these interventions work in a community or health system. Because the focus is on how to make interventions work in real-world settings, the expectation is that the intervention has been demonstrated to be efficacious and effective [7]. Curran [8] describes implementation science versus efficacy/effectiveness research in simplified terms. The intervention or practice or innovation is *The Thing*. Efficacy and effectiveness research investigates whether *The Thing* works. Implementation research studies how to get people and organizations to do *The Thing* and uses implementation strategies or the “stuff we do” to try to help people and organizations to do *The Thing*. Implementation research is interested in outcomes of “how much” and “how well” the people and organizations do *The Thing* [8].

## WHAT IS THE DIFFERENCE BETWEEN IMPLEMENTATION SCIENCE AND QUALITY IMPROVEMENT (QI)?

Implementation science must also be distinguished from quality improvement. Although both have the goal of improving healthcare quality with both using similar techniques and methods for conducting the investigation, there are significant differences. QI tends to be local in nature with problems identified at a local level and results of initiatives often not generalizable to other settings [9].

Implementation science starts with the intervention and investigates how to implement that identified intervention in the health system or community [1], and then, aims to spread the implementation beyond a health system or community. Implementation science like effectiveness/efficacy research has the goal to generalize the results beyond the local context.

## IMPLEMENTATION SCIENCE AND IMPLEMENTATION STRATEGIES

Because implementation science investigates how to get interventions into practice or the community, the research focuses on “implementation strategies” or “the methods or techniques used to enhance adoption, implementation and sustainment of a program or practice” [10]. The study designs used to research these strategies are the same methods used to examine the interventions. Indeed, Procter [10] stated, “the study of implementation strategies should be approached in a similar fashion as evidence-based interventions, for strategies are in fact a type of intervention.”

## WHAT ARE THE THEORIES, MODELS, AND FRAMEWORKS USED IN IMPLEMENTATION SCIENCE?

There are a multitude of theories, models, and frameworks (TMFs) used in implementation science with around 60 TMFs used in studying implementation strategies [11]. Nilsen [4] classified the TMFs into five types:

- Process models
- Determinant frameworks
- Classic theories
- Implementation theories
- Evaluation frameworks

**Figure 1:**
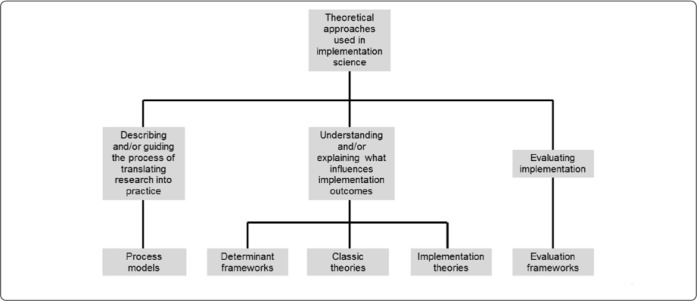
Nilsen [4] classification of implementation science theories, models, and frameworks.

Process models specify steps in the process of translating research into practice. The process models often describe and guide the planning and execution of implementation of an intervention [4]. An example of a process model familiar to hospital librarians working with nurses is the Iowa Model. This model provides nurses with an algorithmic approach to implementing evidence-based interventions starting with identifying an issue to disseminating the results [12]. In 2022, the model expanded to include implementation and sustainability steps [13].

Determinant frameworks guide implementation researchers and practitioners in identifying barriers and facilitators to implementing the intervention. These frameworks aim to understand and/or explain influences on implementation outcomes [4]. The most frequently used determinant framework is the Consolidated Framework for Implementation Research (CFIR). Damschroder et al. [14] developed this framework to help researchers identify barriers and facilitators which can guide assessment, evaluation, and explanations of implementation findings.

Classic theories are theories already used to describe, explain, and predict behavior in individuals and organizations, but are now used to describe, explain, and predict implementation of interventions. These theories come from a variety of fields. From the psychology field, the Theory of Reasoned Action, Social Cognitive Theory, Theory of Planned Behavior, etc. are examples of theories often used in implementation research. Another example from the field of knowledge utilization is Roger's theory of Diffusion of Innovations. This influential theory is considered a classic theory often used to explain intervention adoption [4].

Implementation theories, on the other hand, have been developed specifically to describe, explain, and predict implementation of interventions. Examples of implementation theories include the Implementation Climate, Absorptive Capacity, Organizational Readiness, Normalization Process Theory, etc. The Normalization Process Theory, as an example, identify four determinants that explains the change mechanisms and interrelations needed for implementation [4].

Lastly, evaluation frameworks help determine what could be evaluated for implementation success. As an example, two common frameworks from public health used in implementation science are Reach, Effectiveness, Adoption, Implementation, Maintenance (RE-AIM) and Predisposing, Reinforcing, and Enabling Constructs in Educational Diagnosis and Evaluation-Policy, Regulatory, and Organizational Constructs in Educational and Environmental Development (PRECEDE-PROCEED). These frameworks specify aspects that should be evaluated for when implementing interventions [4]. Proctor et al. [15] developed the Implementation Outcome Framework specifically for implementation research. This framework distinguishes implementation outcomes from services and patient outcomes. Ultimately, the implementation researchers may use many categories of TMFs in combination to answer their question of how to implement an intervention or study an implementation strategy.

## WHAT ARE THE GAPS IN IMPLEMENTATION SCIENCE?

There are still many unanswered questions in implementation science. The largest gap is in the implementation strategies themselves. A major issue is the lack of clarity on the implementation strategies. It is not clear on the individual implementation strategies’ definitions and meaning. This issue includes different strategies having the same definitions or meaning, or one strategy having multiple definitions or meanings [16]. As health sciences librarians and information specialists know, consistent terminology aids in searching and browsing for information. Even with this conceptual confusion, there is little evidence on the effectiveness or adverse consequences of the implementation strategies. The implementation science research arena needs to move beyond identifying barriers and facilitators to studying causal mechanisms of implementation strategies while being aware that different strategies may be more effective in the different phases of implementation or in different contexts [17]. Another concern is that many implementers deploy multiple implementation strategies in addition to the complex interventions. This can confuse the outcomes of the research and make it difficult to evaluate whether the research results are due to the synergistic or antagonistic effects of these multiple implementation strategies and complex interventions [17].

An additional difficulty facing implementation science is the lack of reliable, valid and practical measurements [18], and if there are existing measures, many have not been translated to other languages and cultures. Indeed, much of this research was developed in high-income, English-speaking countries, and there is uncertainty on whether the implementation science research results can be applied in other countries and their local cultural context [19].

Previously, there has been little research about de-implementation and identifying mis-implementation [20]. De-implementation is the process of discontinuing practices that are proven to be ineffective or potentially harmful. Mis-implementation is the mistake of de-implementing effective interventions or the continuation of ineffective interventions [20,21]. There is increasing interest in de-implementation as seen by a recent scoping review searching for frameworks and models that can guide de-implementation [22].

Lastly, implementation science researchers are continuing to investigate new study designs and analytical methods to research implementation strategies and interventions to ensure more rapid implementation of interventions to avoid reproducing the research to practice gap [23].

## WHAT CAN HEALTH SCIENCES LIBRARIANS/INFORMATION SPECIALISTS DO TO SUPPORT IMPLEMENTATION SCIENCE?

Implementation science is a transdisciplinary research methodology taking concepts and methods from many fields. As a transdisciplinary field, health sciences librarians could become part of the research team in implementation science research. It is a matter of how health sciences librarians/information scientists can leverage our knowledge and skills for the research team.

Health sciences librarians can continue to provide our usual services like helping to identify and find implementation-focused research. For example, health sciences librarians could help researchers and practitioners of implementation science identify instruments and questionnaires with an implementation science focus, help researchers perform reviews of the literature to summarize information about implementation strategies and help find reporting guidelines about implementation science research. Health sciences librarians’ involvement in data management, citation analysis, and researcher impact will also help implementation science teams manage information and assess the impact of their research. As the field continues to develop and grow, the variety and inconsistency of terminology leads to barriers in synthesizing and applying findings [24]. Health sciences librarians could help researchers with standardized language and controlled vocabulary development. Health sciences librarians’ skills in gathering and distilling information in a digestible format is also a service we can provide implementation science teams. Health sciences librarians can also provide implementation scientists with information science theories, models, and frameworks that could potentially inform implementation. An example could be Dervin's sensemaking theory which describes the process of information representation and organization to serve a task like decision-making [25]. Implementation researchers can use this classic theory to explain behavior change and implementation due to the healthcare professionals’ assessment of information given to them about the intervention.

## HOW CAN IMPLEMENTATION SCIENCE HELP LIBRARIANS?

Librarians have already been using many quality improvement techniques in assessing, evaluating, and sustaining change in library and information services. The same principle can be used with implementation science in that the theories, models, frameworks, and strategies identified and shown to be effective for implementation of an intervention in turn can be used by librarians who are implementing programs, practices, principles, procedures, products, and/or policies. Health sciences librarians can take a more systematic and rigorous approach to how we implement our interventions such that they can be applied in a variety of library/information settings. An example could be to use evaluation frameworks like RE-AIM to examine the implementation of library programs. Another example could be to use CFIR to identify barriers and facilitators for library programs, and then identify strategies to counteract barriers.

## WHAT IS THE FUTURE OF IMPLEMENTATION SCIENCE?

Given the gaps in implementation science research, but with the importance with implementing effective interventions, more and more researchers from many health fields are now being trained in this area of research. As health sciences librarians and information specialists, health sciences librarians can embrace and become involved in this research and practice area with what health sciences librarians already do to ensure that the clinicians and patients we support receive the best possible care.

Let us start this conversation! Consider joining the Medical Library Association (MLA) translational science or the research caucuses to interact other librarians interested in research and translational science. How else can you envision health sciences librarians’ involvement in implementation science?
